# The High Prevalence of Vitamin D Insufficiency across Australian Populations Is Only Partly Explained by Season and Latitude

**DOI:** 10.1289/ehp.9937

**Published:** 2007-04-17

**Authors:** Ingrid A.F. van der Mei, Anne-Louise Ponsonby, Ola Engelsen, Julie A. Pasco, John J. McGrath, Daryl W. Eyles, Leigh Blizzard, Terence Dwyer, Robyn Lucas, Graeme Jones

**Affiliations:** 1 Menzies Research Institute, University of Tasmania, Hobart, Australia; 2 Murdoch Childrens Research Institute, Melbourne, Australia; 3 Norwegian Institute for Air Research (NILU), Tromsø, Norway; 4 The University of Melbourne, Department of Clinical and Biomedical Sciences, Barwon Health, Geelong, Australia; 5 Queensland Centre for Mental Health Research, The Park Centre for Mental Health, Wacol, Australia; 6 Department of Psychiatry and; 7 School of Biomedical Sciences, University of Queensland, St. Lucia, Australia; 8 National Centre for Epidemiology and Population Health, Australian National University, Canberra, Australia

**Keywords:** 25(OH)D, behavior, latitude, UV index, UVR, vitamin D, vitamin D index

## Abstract

**Background:**

Inadequate sun exposure and dietary vitamin D intake can result in vitamin D insufficiency. However, limited data are available on actual vitamin D status and predictors in healthy individuals in different regions and by season.

**Methods:**

We compared vitamin D status [25-hydroxyvitamin D; 25(OH)D] in people < 60 years of age using data from cross-sectional studies of three regions across Australia: southeast Queensland (27°S; 167 females and 211 males), Geelong region (38°S; 561 females), and Tasmania (43°S; 432 females and 298 males).

**Results:**

The prevalence of vitamin D insufficiency (≤ 50 nmol/L) in women in winter/spring was 40.5% in southeast Queensland, 37.4% in the Geelong region, and 67.3% in Tasmania. Season, simulated maximum daily duration of vitamin D synthesis, and vitamin D effective daily dose each explained around 14% of the variation in 25(OH)D. Although latitude explained only 3.9% of the variation, a decrease in average 25(OH)D of 1.0 (95% confidence interval, 0.7–1.3) nmol/L for every degree increase in latitude may be clinically relevant. In some months, we found a high insufficiency or even deficiency when sun exposure protection would be recommended on the basis of the simulated ultraviolet index.

**Conclusion:**

Vitamin D insufficiency is common over a wide latitude range in Australia. Season appears to be more important than latitude, but both accounted for less than one-fifth of the variation in serum 25(OH)D levels, highlighting the importance of behavioral factors. Current sun exposure guidelines do not seem to fully prevent vitamin D insufficiency, and consideration should be given to their modification or to pursuing other means to achieve vitamin D adequacy.

Most (90–100%) vitamin D is produced endogenously following solar ultraviolet radiation (UVR) of precursors within sun-exposed skin (Holick 1994). Only a few foods, such as oily fish, contain significant amounts of vitamin D naturally (Holick 1994). Serum 25-hydroxyvitamin D [25(OH)D] is widely recognized as the best measure of vitamin D status (Holick 2004a), and reflects UVR exposure and vitamin D intake of the previous few months (Vieth 1999). In the absence of dietary fortification, vitamin D intake in Australia is low, varying from 1.2 to 2.6 μg/day (Nowson and Margerison 2002). This is much lower than the recommended dietary intake (5 μg/day for those 0–50 years of age, 10 μg/day for those 51–70 years, 15 μg/day for those > 70 years) (Commonwealth of Australia 2006) or the estimated intake of 10–15 μg/day required to obtain serum 25(OH)D levels of 50 nmol/L (Dawson-Hughes et al. 2005). Vitamin D status is often defined by serum levels of 25(OH)D as follows: vitamin D deficiency, 25(OH)D < 25 nmol/L; vitamin D insufficiency, 25(OH)D of 25–50 nmol/L; and optimal status, 25(OH)D > 50 nmol/L (Nowson and Margerison 2002; Vieth et al. 2001). Recent evidence suggests that the optimal serum 25(OH)D levels may be even higher, > 80 nmol/L (Dawson-Hughes et al. 2005; Hollis 2005).

A balance is required in the amount of personal UVR exposure. Excessive sun exposure has been associated with increased risk of cutaneous malignant melanoma, non-melanoma skin cancers, and some cataracts (Lucas et al. 2006); however, low UVR exposure can result in vitamin D deficiency or insufficiency. It has been well established that vitamin D is essential to bone health, with low levels associated with rickets, osteoporosis, and osteomalacia (Holick 2004b). Vitamin D insufficiency causes muscle weakness and may contribute to falls in the elderly, which—when associated with osteoporosis—increases the likelihood of fractures. However, more recent evidence indicates that vitamin D insufficiency might also be associated with diseases such as colorectal cancer, prostate cancer, multiple sclerosis (MS), type 1 diabetes, cardiovascular diseases, and tuberculosis (Hughes et al. 2004; Hypponen et al. 2001; Luscombe et al. 2001; van der Mei et al. 2003; Zittermann 2003).

The “sun smart” public health messages, aimed at reducing skin cancer, have been successful, partly because of their simplicity. However, there is increasing awareness that any simple public health message of sun avoidance may cause harm by increasing vitamin D insufficiency (Grant et al. 2005; Sinclair 2006). A more balanced approach is now being taken, and new public health messages are being developed that provide a more tailored approach concerning factors such as latitude, time of year, skin type, and age (Holick 2004b; Samanek et al. 2006; Working Group of the Australian and New Zealand Bone and Mineral Society, Endocrine Society of Australia, and Osteoporosis Australia 2005). Tools, such as the UV Index [based on a weighting of UV irradiances that produce erythema (i.e., sunburn)] are being used to identify the appropriate times of the year or day that sun protection is most important (Sinclair 2006). The World Health Organization (WHO 2007) and the Cancer Council Australia (2007) currently recommend that protective measures should be taken (e.g., use of a hat, sunscreen, or sunglasses; seek shady areas) when the UV index is ≥ 3. In addition, recommended solar UVR exposure periods to maintain vitamin D sufficiency have been calculated at different locations based on ambient UVR (assuming sufficiency is maintained by one-third of a minimal erythemal dose of sunlight for an individual with moderately fair skin who exposes 15% of the body (e.g., face, arms, hands) (Samanek et al. 2006). This information is important, but a stronger evidence base is required on the actual serum 25(OH)D status in different regions and by season, reflecting interindividual differences in sun exposure behaviors and other factors, rather than the predicted serum 25(OH)D status from ambient UVR alone.

We pooled population-based samples of individuals < 60 years of age with serum measurements of 25(OH)D across a broad latitudinal range in Australia [southeast Queensland, 27°S; the Geelong region (specifically the Barwon Statistical Division), 38°S; and Tasmania, 43°S] (McGrath et al. 2001b; Pasco et al. 2001; van der Mei et al. 2007). We also included new data on a large population-based sample from Tasmania [the Tasmanian Older Adult Cohort (TasOAC) study]. In the present study, we aimed to *a*) examine the prevalence of vitamin D deficiency and insufficiency by season at each of the three locations; *b*) examine differences in the seasonal variation between the three locations in terms of the timing of the peak and the trough, the magnitude of the amplitude of the seasonal variation and the estimated mean, peak, and trough 25(OH)D values; and *c*) examine to what extent actual serum 25(OH)D levels are predicted by season, latitude, and the maximum daily duration of vitamin D synthesis in human skin, and vitamin D effective daily dose.

## Methods

### Participants

In this study we used data from three published studies (McGrath et al. 2001b; Pasco et al. 2001; van der Mei et al. 2007) and the TasOAC study, covering three regions and including subjects in varying age ranges. All of these studies included participants < 60 years of age and provided a good sample of young and middle-aged adults.

#### Southeast Queensland

A case–control study on psychosis was conducted in southeast Queensland from 1997 to 1999 and included 310 cases and 303 controls (McGrath et al. 2001a, 2002). All subjects provided written informed consent, and the study was approved by the Wolston Park Hospital Institutional Ethics Committee. For the present study, we selected only those subjects < 60 years of age (167 women and 211 men) for whom serum 25(OH)D levels were available.

#### Geelong region

In the Geelong region (in southern Victoria), an age-stratified, random, population-based sample of women (*n* = 1,494) was recruited from Commonwealth Electoral Rolls (1993–1997), with a response rate of 77.1% (Henry et al. 2000; Pasco et al. 2000). Of the subjects of the original study, 99% were white, and none were shrouded for religious reasons. Written informed consent was obtained from all participants, and the project was approved by the Barwon Health Human Research and Ethics Committee. For the present study, we used data only from the women < 60 years of age if they were free from exposure to drugs and diseases known to influence calcium metabolism (*n* = 561).

#### Tasmania

In Tasmania, control participants from the Tasmanian MS case–control study (1999–2001) (van der Mei et al. 2003) and participants from the TasOAC study (2002–2004) were included. The controls from the Tasmanian MS case–control study (van der Mei et al. 2003) (*n* = 272; response rate 76%) were randomly drawn from the Tasmanian Electoral Roll and matched on sex and birth year to prevalent MS cases < 60 years of age. Data from 174 women and 88 men with serum 25(OH)D were available. The TasOAC study included participants 50–80 years of age randomly selected from the Tasmanian Electoral Roll (response rate 54%); all participants < 60 years of age (258 women and 211 men) were included. Written informed consent was obtained from all participants, and the projects were approved by the Human Research Ethics Committee of the Royal Hobart Hospital.

### Measurements and simulations

For the present study, the following data were available at an individual level for all of the studies: serum 25(OH)D, date of serum sample collection, date of birth, and sex. In all studies, serum 25(OH)D was measured using a radio-immunoassay kit (DiaSorin, Stillwater, MN, USA) (McGrath et al. 2001b; Pasco et al. 2001; van der Mei et al. 2007). The intraassay and interassay precisions of these assays are 6% and 15%, respectively. Of the four studies, only participants in the TasOAC study in Tasmania provided information on the amount of time spent in the sun during weekends and holidays in the past winter and summer (< 1 hr/day, 1–2 hr/day, 2–3 hr/day, 3–4 hr/day, or > 4 hr/day).

For each of the three locations, we used the VitD methodology of Engelsen et al. (2005) to estimate maximum daily duration of vitamin D synthesis in human skin, vitamin D effective daily dose, and the UV index for each day in the years that the four studies were conducted. Maximum daily duration of vitamin D synthesis in human skin is the time per day in hours in which UVR exceeds the threshold required to produce vitamin D (Brustad et al. 2004; Webb et al. 1988). Vitamin D effective daily dose is the daily dose of UVR wavelengths relevant to the conversion of 7-dehydrocholesterol into previtamin D in the skin, based on the vitamin D action spectrum (MacLaughlin et al. 1982). This is important because the action spectrum to produce previtamin D is different from the action spectrum that produces erythema (sunburn).

First, using the method of MacLaughlin et al. (1982), we established a biologically effective UV dose rate for photoconversion of 7-dehydrocholesterol to previtamin D in skin by integrating the measured UV surface irradiances weighted by the relative efficiencies for converting 7-dehydrocholesterol to previtamin D. Then, the biologically effective dose was integrated over a full day to estimate the vitamin D effective daily dose. The UV index (WHO 2002) gives an indication of the UV intensity at solar noon using the action spectrum to produce erythema. The daily values were calculated without rounding to nearest integer, but the monthly mean values were rounded to the nearest integer.

To estimate UV irradiances, taking ozone levels into account, we used the VitD methodology of Engelsen et al. (2005). Satellite ozone data from the Total Ozone Mapping Spectrometer (TOMS) were used for Brisbane (for southeast Queensland), Aspendale (for the Geelong region), and Hobart (for Tasmania). From 25 November 1994 to 24 July 1996, no TOMS satellite instruments were in operation. Because the Geelong study was conducted during this period, the TOMS ozone series for the Geelong region was complemented with Dobson ozone measurements from Melbourne Airport. All remaining total ozone data gaps were filled using linear interpolation. We assumed a dry concrete surface at sea level and a cloudless atmosphere with a surface visibility of 25 km. All other remaining model parameters used for the simulations of UV radiation were fixed and identical to those reported by Engelsen et al. (2005).

### Statistical analysis

Because the sample in the Geelong region consisted of women only, most analyses were restricted to women; however, in some instances, comparisons were made with men. Tests for differences of the mean age by location or serum 25(OH)D levels by sex were performed using analysis of variance (ANOVA). In southeast Queensland, half the sample had a diagnosis of psychosis, but we found no difference in mean 25(OH)D between the groups with and without that diagnosis (*p* = 0.98) (McGrath et al. 2001b). The prevalence of vitamin D deficiency or insufficiency was calculated using commonly used cut-points (≤ 25 nmol/L and ≤ 50 nmol/L, respectively) (Nowson and Margerison 2002; Vieth et al. 2001). We also used ≤ 60 nmol/L and ≤ 80 nmol/L as cut-points, because there is an increasing debate whether optimal serum 25(OH)D levels might be > 50 nmol/L (Dawson-Hughes et al. 2005; Hollis 2005). We used logistic regression to examine whether the prevalence of vitamin D insufficiency or deficiency differed by latitude by including the actual latitude of each of the regions as a predictor. We adjusted for season by including binary (0, 1) terms for three of the four seasons (summer, autumn, winter, and spring).

To model the seasonal variation of serum 25(OH)D, we fitted a sinusoidal model to the actual serum 25(OH)D levels and the month the sample was taken (*t*):


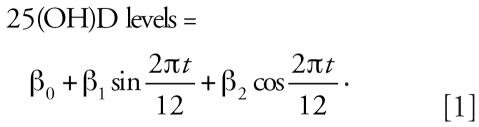


With the same method we modeled the seasonal variation of simulated daily duration of vitamin D synthesis and simulated vitamin D effective daily dose. We used the ANOVA *F*-test to determine whether the seasonal variation was significant, and we calculated the amplitude using the formula


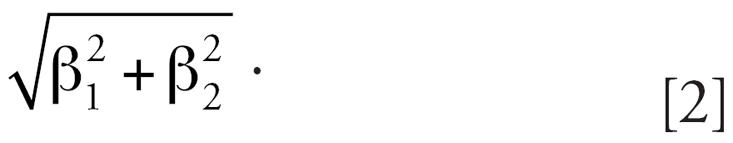


The peak and trough were found by taking the first derivative of the sinusoidal function and solving for the value of *t* for which the first derivative was zero. This gave the formula


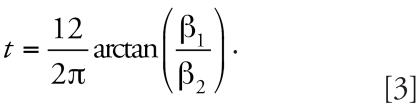


For the test for trend of the mean serum 25(OH)D levels by location, we tested the significance of a covariate taking latitude values for each location (southeast Queensland = 27, Geelong = 38, Tasmania = 43). For the test for trend by location of the estimated mean maximum daily duration of vitamin D synthesis and vitamin D effective daily dose, we followed the same procedure but allowing variation in the seasonal patterns between the three locations. For tests for trend of the estimated amplitude for serum 25(OH)D, maximum daily duration of vitamin D synthesis, and vitamin D effective daily dose, we used meta-regression methods with precision weighting of the point estimates and adjustment of SEs using the procedure described by Greenland (1998). To compare the seasonal pattern in 25(OH)D in men and women, we added a sex interaction for each of the sine and cosine components of the sinusoidal model, and tested the improvement in fit using a partial *F*-test. To examine predictors of 25(OH)D, we calculated Pearson correlation coefficients or used linear regression. For these analyses, 25(OH)D values were transformed to reduce skewness using a square root transformation. A logarithmic transformation produced similar results (data not shown). With linear regression, tests for interaction were conducted using the coefficient and SE of a product term.

## Results

Table 1 shows that the sample of women in the Geelong region was younger than the samples in southeast Queensland (*p* = 0.02) and Tasmania (*p* < 0.01). Serum 25(OH)D levels were not strongly associated with age in any of the three samples (southeast Queensland, *r* = −0.02, *p* = 0.74; Geelong region, *r* = −0.08, *p* = 0.07; Tasmania, *r* = 0.01, *p* = 0.81). As expected, mean levels of serum 25(OH)D were lower in women than in men (southeast Queensland: women, 67.0 nmol/L and men 72.2 nmol/L, *p* = 0.06; Tasmania: women, 51.1 nmol/L and men, 55.2 nmol/L, *p* < 0.01).

### Prevalence of vitamin D insufficiency and deficiency by location and by season

**Figure 1:**
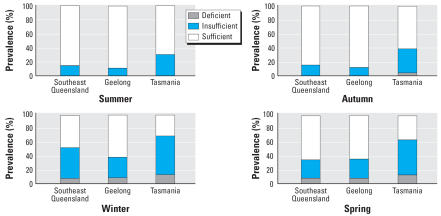
Prevalence of vitamin D deficiency (≤ 25 nmol/L), vitamin D insufficiency (26–50 nmol/L), and vitamin D sufficiency (> 50 nmol/L) for women < 60 years years of age in southeast Queensland (latitude 27°S), Geelong (latitude 38°S), and Tasmania (41–43°S) by season.

Figure 1 shows the prevalence of vitamin D deficiency and insufficiency in women by season. Irrespective of their location across Australia, vitamin D insufficiency was common in winter and spring. For example, the prevalence of levels ≤ 50 nmol/L in winter/spring was 40.5% for southeast Queensland, 37.4% for the Geelong region, and 67.3% for Tasmania. The prevalence of deficiency (< 25 nmol/L) in women was also highest in winter and spring (7.1% in southeast Queensland, 7.9% in the Geelong region, and 13.0% in Tasmania). If we define vitamin D insufficiency as levels ≤ 60 nmol/L or ≤ 80 nmol/L, respectively, then the prevalence in winter/spring would be as follows: southeast Queensland 54.8% and 82.1%; Geelong region, 50.2% and 74.0%; and Tasmania, 83.7% and 97.0%.

In this sample pooled over three locations, vitamin D insufficiency (*p* < 0.01, adjusted for season) and vitamin D deficiency (*p* = 0.05, adjusted for season) increased, on average, with increasing latitude.

In the TasOAC study in Tasmania, data were also available on the amount of time participants spent in the sun during weekends and holidays in the past winter (here termed “winter sun exposure”). People who reported more winter sun exposure had higher levels of serum 25(OH)D in winter (*r* = 0.28, *p* < 0.01) and spring (*r* = 0.25, *p* = 0.02); the prevalence of vitamin D insufficiency and deficiency in winter/spring was also lower among those who had most sun exposure: Vitamin D insufficiency and deficiency were 42.9% (12/28) and 7.1% (2/28), respectively, among those who reported on average > 4 hr/day of sun, and 74.6% (50/67) and 19.4% (13/67) among those who reported on average < 1 hr/day of sun.

### Monthly prevalence of vitamin D insufficiency and deficiency compared with UV index

**Figure 2:**
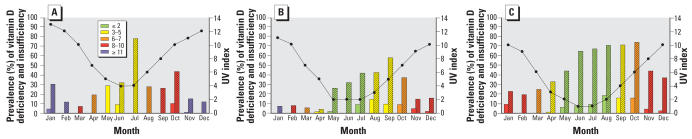
Prevalence of vitamin D deficiency (≤ 25 nmol/L; solid bars) and insufficiency (≤ 50 nmol/L; stippled bars) and the UV index (•—•) on a cloudless day by month of the year in (*A*) southeast Queensland, (*B*) the Geelong region, and (*C*) Tasmania. Colors of the bars represent the current sun protection recommendations based on the UV index: green (UV index ≤ 2), can safely stay outdoors with minimal protection; yellow (UV index 3–5), wear hat, sunscreen, sunglasses, seek shady areas; orange (UV index 6–7), see yellow and stay indoors between 1000 and 1400 hours (1100–1500 hours daylight saving time); red (UV index 8–10), see orange and stay indoors as much as possible; blue (UV index ≥ 11), see red.

Figure 2 shows the monthly prevalence of vitamin D insufficiency and deficiency as well as the average monthly UV index, simulated for cloudless conditions. The colors of the bars represent the current sun protection recommendations based on the UV index for a cloudless day: green indicates that the UV level is low and no or minimal protection is required, whereas the other colors indicate that sun protection should be used. In southeast Queensland, there is a high prevalence of vitamin D insufficiency in July, which is midwinter; however, UV protection is still recommended at this time of year. In Geelong, there is substantial vitamin D insufficiency and deficiency during winter and spring, and for a large part of that time sun protection is recommended. In Tasmania, vitamin D insufficiency and deficiency seems to be common for a large part of the year, including the months May–August, when generally no protection is recommended.

### Seasonal variation in serum 25(OH)D levels, maximum daily duration of vitamin D synthesis, and vitamin D effective daily dose

#### Serum 25(OH)D

**Figure 3:**
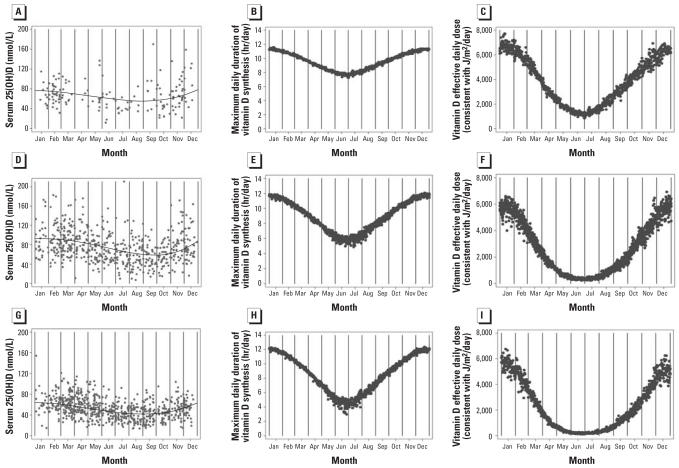
Seasonal variation in actual serum 25(OH)D levels and predicted serum 25(OH)D (solid line) (*A, D, G*), simulated maximum daily duration of vitamin D synthesis in human skin (*B, E, H*), and simulated vitamin D effective daily dose (*C, F, I*) in southeast Queensland (*A–C*), the Geelong region (*D–F*), and Tasmania (*G–I*).

At each location, there was significant seasonal variation in 25(OH)D levels among women (*p* < 0.01) (Figure 3A,D,G). Surprisingly, the Geelong region had the highest mean 25(OH)D levels (*p* < 0.01 compared with southeast Queensland; *p* < 0.01 compared with Tasmania) and the strongest amplitude (Table 2). Southeast Queensland had the least seasonal variation in serum 25(OH)D. As expected, Tasmania had the lowest mean 25(OH)D level. The amplitude of seasonal variation in men in southeast Queensland was similar to that of the women (mean 72.2 nmol/L, amplitude 10.6 nmol/L, peak 80.5 nmol/L in early January, trough 59.6 nmol/L in early July). In Tasmania, the amplitude was greater for men than for women (*p* = 0.03; mean 55.2 nmol/L, amplitude 16.2 nmol/L, peak 70.5 nmol/L in mid-February, trough 38.2 nmol/L in mid-August).

#### Maximum daily duration of vitamin D synthesis

The predicted seasonal variation of simulated maximum daily duration of vitamin D synthesis in human skin is shown in Figure 3B, 3E, and 3H. Our analyses show that southeast Queensland had the greatest mean duration per day when UVR was such that vitamin D production could occur (Table 3). In addition, southeast Queensland had the least seasonal variation over the year (amplitude) in hours per day of vitamin D production (Table 3). Tasmania had the lowest mean duration per day when vitamin D production could occur, but the greatest seasonal variation over the year and the highest number of hours of vitamin D production in summer (peak). The time of the year of the peaks and troughs were the same for all locations (Table 3).

#### Vitamin D effective daily dose

Table 3 and Figure 3C, 3F, and 3I show the seasonality in the daily dose of UVR effective for vitamin D production at each location, based on the vitamin D action spectrum for the conversion of 7-dehydrocholesterol into previtamin D. Although Tasmania had the highest peak for daily hours of vitamin D production in summer compared with the other regions, the vitamin D effective daily dose in that location was the lowest of the three regions in summer (Table 3, Figure 3C,F,I). This is consistent with the known lower intensity of UVR per hour in Tasmania.

### Multivariable analysis of serum 25(OH)D levels

#### Latitude and season

For the total sample of women, we examined the contribution of season by fitting a sinusoidal model to square root–transformed 25(OH)D levels and the month the serum sample was collected. Season explained 13.6% of the variation in 25(OH)D levels. We noted above that the effect of season was less pronounced for southeast Queensland than for Geelong and Tasmania, but a test of difference of the seasonal pattern by latitude was not significant (*p* = 0.21 for interaction). A higher latitude was significantly associated with lower serum 25(OH)D (*p* < 0.01). Although it only explained 3.9% of the variation in 25(OH)D, the clinical contribution of higher latitude seems substantial because serum 25(OH)D decreased on average by 1.0 nmol/L (95% confidence interval, 0.7–1.3 nmol/L) for every degree increase in latitude. Also, after taking season into account, latitude remained significantly associated with serum 25(OH)D (*p* < 0.01). This model, including latitude and season, predicted 16.5% of the variation in 25(OH)D levels.

#### Maximum daily duration of vitamin D synthesis and vitamin D effective daily dose

We found a high correlation (*r* = 0.92) between simulated maximum daily duration of vitamin D synthesis in human skin and vitamin D effective daily dose. Maximum daily duration of vitamin D synthesis explained 7.0% of the variation in serum 25(OH)D levels, and vitamin D effective daily dose explained 7.8%. Shifting the data so the estimated peaks and troughs were aligned with the estimated peak and trough of serum 25(OH)D levels at each location made a substantial difference. After alignment, maximum daily duration of vitamin D synthesis explained 14.5% of the variation in serum 25(OH)D levels, and vitamin D effective daily dose explained 14.8%. The lag time between the estimated peak of maximum daily duration of vitamin D synthesis and the estimated peak in serum 25(OH)D was 26 days for southeast Queensland, 53 days for the Geelong region, and 47 days for Tasmania. For vitamin D effective dose, the lag times were 21 days for southeast Queensland, 49 days for the Geelong region, and 43 days for Tasmania.

Interestingly, a model including maximum daily duration of vitamin D synthesis with season and latitude did not explain much more of the variation of serum 25(OH)D (17.1%) than a model including season and latitude alone (16.5%). In line with this, after adjustment for maximum daily duration of vitamin D synthesis and latitude, there remains a seasonal pattern (*p* < 0.01), but it is diminished. Similarly, after adjustment for the seasonal pattern and latitude, the association between maximum daily duration of vitamin D synthesis and serum 25(OH)D is significant (*p* < 0.01) but diminished. This indicates that both variables partly capture the same information. The same was true for the analyses using vitamin D effective daily dose.

## Discussion

In three population-based samples of women < 60 years of age across Australia [southeast Queensland (27°S), Geelong region (38°S), and Tasmania (43°S)], vitamin D insufficiency was common in winter and spring. Season was a strong determinant of vitamin D status. Simulated maximum daily duration of vitamin D synthesis and vitamin D effective daily dose were also important predictors of serum 25(OH)D levels after aligning the seasonal patterns of those variables with seasonal pattern of serum 25(OH)D. The contribution of latitude was also evident but of lower magnitude. In some months when sun exposure protection would be recommended based on the simulated UV index, there was a high insufficiency or even deficiency.

Many studies assessing vitamin D status have been conducted in groups at high-risk of vitamin D deficiency, such as the elderly, infants, or veiled women. These data cannot be generalized to the entire adult Australian population. In this study we examined population-based samples of women < 60 years of age in three locations covering a broad latitudinal range. The limitations of this study are that the three populations were recruited with their own eligibility criteria, which could have resulted in some selection bias. For example, the vitamin D insufficiency in the Geelong region was lower than expected, on the basis of its latitude. Although we cannot rule out selection bias, the sample of women in the Geelong region was recruited from the Commonwealth Electoral Roll with a high response rate, and the sample was relatively large. In addition, we could examine the effect of latitude and season on 25(OH)D, but we could not directly determine to what extent 25(OH)D levels were determined by other factors such as skin type, dietary intake, sun behavior, and sun avoidance behavior. A large percentage of the study participants considered here were Caucasian. In Geelong, 99% were of European descent, and 81% reported having a skin type that sunburned easily or moderately easily (Pasco et al. 2001). In Tasmania, all participants were Caucasian (van der Mei et al. 2003). However, even within Caucasians, skin type could have had an influence on vitamin D status (van der Mei et al. 2007). The contribution of dietary intake of vitamin D to serum 25(OH)D levels was probably small because the intake of vitamin D in Australia is generally low (Nowson and Margerison 2002). Indeed, in the Geelong region dietary intake was low (1.2 μg/day), and only 7.9% regularly used vitamin D–containing supplements (Pasco et al. 2001). In Tasmania, 8.1% of participants used vitamin D–containing supplements; no association was observed between serum 25(OH)D and the use of vitamin D–containing supplements or intake of fish, milk, eggs, or meat (van der Mei et al. 2007). For the location-specific simulations of maximum daily duration of vitamin D synthesis, vitamin D effective daily dose, and UV index, changes in ambient conditions (clouds and aerosols) were not taken into account. Therefore, our simulated values can be expected to be higher than the true values.

Our data confirm that season is an important predictor of serum 25(OH)D levels, as previously reported by Webb et al. (1988). Season is known to be associated with ambient ultraviolet radiation; however, it can also reflect changes in outdoor behavior and amount of clothing worn. In an *in vitro* study conducted in Johannesburg, South Africa (latitude 26°S), Pettifor et al. (1996) observed no seasonal variation throughout the year in the formation of previtamin D_3_ and vitamin D_3_ under the influence of ambient ultraviolet radiation. However, a clear seasonal pattern in 25(OH)D levels has been shown at an individual level (Pettifor et al. 1978). This suggests that the seasonal variation at an individual level [shown in an elderly population in Johannesburg by (Pettifor et al. 1978)] is a consequence of the increased clothing worn and the decreased time spent outdoors during winter, rather than decreased vitamin D–effective UVR reaching the Earth. Although season was a strong predictor of serum 25(OH)D, latitude was not as important as might have been expected based on the knowledge that ambient ultraviolet radiation levels (and particularly UVB) generally decrease with increasing latitude (*r* = −0.96 for annual ambient UVR) (Gies et al. 1999). However, the effect of latitude was significant, and its contribution is likely to be clinically relevant with serum 25(OH)D levels, on average decreasing 1.0 nmol/L (95% confidence interval, 0.7–1.3 nmol/L) for every degree increase in latitude.

A latitudinal gradient in 25(OH)D levels has been found within a number of countries such as France (43–55°N) (Chapuy et al. 1997) and Argentina (26–55°S) (Oliveri et al. 2004). Interestingly, across countries in Europe, a latitudinal gradient in the opposite direction has been demonstrated among healthy elderly individuals, with a lower prevalence of vitamin D insufficiency in northern Europe compared with southern Europe (Lips et al. 2001). One explanation could be that countries where a negative latitudinal gradient in 25(OH)D levels was observed might be more homogeneous in regard to ethnicity and living and dietary habits (Oliveri et al. 2004). Across countries, cultural differences in constitutional skin color, outdoor behavior, diet, and clothing would have played a role, as well as vitamin D supplementation policies (Scharla 1998).

Location-specific estimates of ambient UVR important for the production of vitamin D (maximum daily duration of vitamin D synthesis and vitamin D effective daily dose) were also a strong determinant of serum 25(OH)D after aligning the seasonal patterns of those variables with the seasonal pattern of serum 25(OH)D, although they seemed to capture similar information as the seasonal variation in 25(OH)D. A lag period between the maximum UV dose and the peak 25(OH)D levels has been described previously (Lucas et al. 2005; Pasco et al. 2004). Here, using simple sinusoidal models, we observed that the lag time was approximately twice as long for Tasmania as for southeast Queensland. This variation in the number of days by which serum 25(OH)D lagged behind maximum daily duration of vitamin D synthesis and vitamin D effective daily dose may reflect behavioral factors (e.g., amount of clothing worn), the biological lag between UVR exposure and 25(OH)D synthesis, and/or other location-specific variables (e.g., actual level of vitamin D effective daily dose).

Importantly, we found a concerning high prevalence of vitamin D insufficiency in winter and spring, irrespective of the location within Australia. In winter/spring, the prevalence of insufficiency (≤ 50 nmol/L) was 40.5% in southeast Queensland, 37.4% in the Geelong region, and 67.3% in Tasmania. Thus, even residence in a sunny climate, such as southeast Queensland (27°S), did not prevent vitamin D insufficiency. The same was found in a healthy adult population in south Florida (USA; 25°N), where 40% of the women and 38% of the men had serum 25(OH)D levels < 50 nmol/L at the end of winter (Levis et al. 2005). These authors thought that avoidance of sun exposure because of the heat and increased awareness of the risk of developing skin cancer were the underlying reasons for the high prevalence of insufficiency.

In Tasmania (43°S), time in the sun was a predictor of 25(OH)D; although higher levels of sun exposure are associated with better vitamin D status, the prevalence of vitamin D insufficiency in winter/spring was still high (43%) among the subgroup of people that were most sun seeking (time in the sun > 4 hr/day during weekends and holidays). This suggests that, in winter and spring, the combination of low ambient UVR and an increased amount of clothing worn makes it difficult to achieve an adequate vitamin D status (> 50 nmol/L) without a high dietary intake of vitamin D or vitamin D supplementation. The current vitamin D and adult bone health position statement in Australia and New Zealand indicates that in winter in the southern states, vitamin D levels may be maintained by approximately 2–3 hr of sunlight exposure accumulated over a week to the face, arms, and hands or equivalent surface area (Working Group of the Australian and New Zealand Bone and Mineral Society, Endocrine Society of Australia, Osteoporosis Australia 2005). Our data suggest that recommended exposure should be reconsidered and/or that vitamin D supplementation might be required.

The question of what an “optimal” level or range of serum 25(OH)D might be remains difficult to answer, but suggestions have been made that the optimal serum 25(OH)D levels may be > 50 nmol/L (Dawson-Hughes et al. 2005; Hollis 2005). In our study samples, > 50% of participants would be vitamin D insufficient in winter if the cut-off were 60 nmol/L, and > 70% if the cut-off were 80 nmol/l. Furthermore, even in summer, a high percentage of the population would be insufficient for vitamin D if those higher cut-off points were used.

A national cohort study of Caucasian 45-year-old subjects in the United Kingdom has also reported a high prevalence of hypo-vitaminosis D in the general population, warranting action at a population, not individual level (Hypponen and Power 2007). Our data indicate that even in Australia, a location with higher ambient UVR, similar problems exist. Our study findings indicate that sun exposure protection is recommended in Australia in some months or regions where vitamin D insufficiency is high or where vitamin D deficiency exists. A full discussion of the potential solution to this problem, taking into account other health effects of UVR such as skin cancer, are beyond the scope of this article; however, policy strategies requiring consideration include *a*) the greater promotion of safe sun exposure while avoiding adverse UVR effects; *b*) adequate vitamin D supplementation; and *c*) vitamin D fortification of foods. With regard to the first option, our findings that maximum daily duration of vitamin D synthesis and vitamin D effective daily dose were important predictors of serum 25(OH)D indicate that the use of a vitamin D index could be a valuable additional tool (Kimlin 2004). Using a vitamin D index in addition to the UV index for recommending sun exposure to achieve and/or maintain vitamin D adequacy, within the range of exposure that avoids adverse UVR effects, might be useful because the UV index is weighted by UVB and UVA wavelengths that produce erythema (sunburn), which is different from the UVB-only action spectrum for vitamin D photoproduction. The present study also particularly emphasizes that living at low latitude does not prevent vitamin D insufficiency and that behavior (amount of time outside, amount of clothing) is important. Education should be provided on these issues. In addition, in Tasmania, a state with a high level of vitamin D insufficiency, we had data available on personal sun exposure. The prevalence of vitamin D insufficiency in winter and spring was high even among those who reported high levels of sun exposure, suggesting that vitamin D levels should be monitored and that increased dietary intake of vitamin D, either through supplements or fortification, should also be considered.

In conclusion, we found that vitamin D insufficiency was common in winter and spring in three locations in Australia, covering a latitude of 27°S to 43°S. Season was more important than latitude in determining serum 25(OH)D. However, both only explained less than one-fifth of the variation in 25(OH)D levels, highlighting the importance of behavioral factors. Simulated maximum daily duration of vitamin D synthesis and vitamin D effective daily dose were also important predictors of serum 25(OH)D, indicating that a location-specific indicator of vitamin D production, such as the vitamin D index, could be useful for recommendations for the achievement and maintenance of vitamin D adequacy. Current sun exposure practices and dietary intake do not seem to fully prevent vitamin D insufficiency and deficiency, and consideration should be given to modification of sun exposure advice or pursuing other means to achieve vitamin D adequacy.

## Figures and Tables

**Table 1:** Age and season of blood sampling in population-based samples of men and women < 60 years.

	Southeast Queensland		Geelong region	Tasmania	
	Females No. (%)	Males No. (%)	Females No. (%)	Females No. (%)	Males No. (%)
Age (years)					
< 20	6 (3.6)	7 (3.3)	0 (0)	0 (0)	0 (0)
20–29	34 (20.4)	41 (19.4)	162 (28.9)	17 (3.9)	1 (0.3)
30–39	38 (22.8)	65 (30.8)	177 (31.6)	45 (10.4)	21 (7.1)
40–49	49 (29.3)	61 (28.9)	144 (25.7)	67 (15.5)	34 (11.4)
50–59	40 (24.0)	37 (17.5)	78 (13.9)	303 (70.1)	242 (81.2)
Total	167	211	561	432	298
Season serum sample collected					
Summer (Dec–Feb)	55 (32.9)	44 (20.9)	121 (21.6)	57 (13.2)	49 (16.4)
Autumn (Mar–May)	28 (16.8)	63 (29.9)	175 (31.2)	167 (38.7)	114 (38.3)
Winter (Jun–Aug)	27 (16.2)	24 (11.4)	136 (24.2)	103 (23.8)	77 (25.8)
Spring (Sep–Nov)	57 (34.1)	80 (37.9)	129 (23.0)	105 (24.3)	58 (19.5)
Total	167	211	561	432	298

**Table 2:** Characteristics of the seasonal variation^a^ in serum 25(OH)D levels in population-based samples of women < 60 years of age in southeast Queensland, the Geelong region, and Tasmania.

		Estimated				
	Mean (nmol/L)	Amplitude^b^ (nmol/L)	Peak (nmol/L)	Month peak	Trough (nmol/L)	Month trough
Southeast Queensland	67.0	10.34	75.3	Early January	54.6	Early July
Geelong region	75.5	17.7	92.5	Early February	57.1	Early August
Tasmania	51.1	10.9	62.1	Early February	40.3	Early August
Test for trend	*p* < 0.01	*p* = 0.15				

a A sinusoidal model was applied to the actual 25(OH)D levels and the month the serum sample was taken.

b Half the difference between the estimated peak and trough.

**Table 3:** Characteristics of the estimated seasonal variation^a^ in simulated maximum daily duration of vitamin D synthesis in human skin and simulated vitamin D effective daily dose in southeast Queensland, the Geelong region, and Tasmania.

	Mean	Amplitude^b^	Peak	Month peak	Trough	Month trough
Maximum daily duration of vitamin D synthesis (hr/day)						
Southeast Queensland	9.6	1.7	11.3	Mid-January	7.9	Mid-July
Geelong region	9.0	2.8	11.8	Mid-January	6.1	Mid-July
Tasmania	8.6	3.5	12.1	Mid-January	5.1	Mid-July
Test for trend	*p* < 0.01	*p* < 0.01				
Vitamin D effective daily dose (consistent with J/m^2^/day)						
Southeast Queensland	3,848	2,639	6,487	Mid-January	1,209	Mid-July
Geelong region	2,642	2,687	5,330	Mid-January	−45	Mid-July
Tasmania	2,220	2,538	4,758	Mid-January	−319	Mid-July
Test for trend	*p* < 0.01	*p* < 0.01				

a A sinusoidal model was applied to the simulated maximum daily duration of vitamin D synthesis or the simulated vitamin D effective daily dose and the month of the year; data were included for complete years in which each study was completed.

b Half the difference between the estimated peak and trough.
